# From efficacy to effectiveness: child and adolescent eating disorder treatments in the real world (Part 2): 7-year follow-up

**DOI:** 10.1186/s40337-022-00535-8

**Published:** 2022-02-05

**Authors:** Catherine S. Stewart, Julian Baudinet, Alfonce Munuve, Antonia Bell, Anna Konstantellou, Ivan Eisler, Mima Simic

**Affiliations:** 1 Maudsley Centre for Child and Adolescent Eating Disorders (MCCAED), Maudsley Hospital, De Crespigny Park, Denmark Hill, London, SE5 8AZ UK; 2 Institute of Psychiatry, Psychology and Neuroscience (IoPPN), King’s College London, 16 De Crespigny Park, Denmark Hill, London, SE5 8AF UK

**Keywords:** Child, Adolescent, Anorexia nervosa, Bulimia nervosa, Family therapy for anorexia nervosa, Family therapy for bulimia nervosa, Family based treatment

## Abstract

**Background:**

Eating disorders are often characterised as disabling, chronic or relapsing conditions with high mortality rates. This study reports follow-up outcomes for patients seen at the Maudsley Centre for Child and Adolescent Eating Disorders (MCCAED), whose end of treatment outcomes are reported in a separate paper.

**Methods:**

Three-hundred-and-fifty-seven former patients, who received evidence-based treatment for an eating disorder as a child or adolescent in MCCAED between 2009 and 2014 were eligible to participate. Current contact information was available for 290, of whom 149 (51.4%) consented to follow-up. Participants were sent links to online questionnaires, with additional demographic information extracted from medical records. Descriptive analyses of key socioeconomic and health outcomes were performed on data collected.

**Results:**

Mean length of follow-up was 6 years 11 months. Ten (6.7%) participants reported a current diagnosis of an eating disorder at follow-up. The great majority reported no (63.8%) or minimal (26.8%) interference from eating disorder difficulties. More than half (53.6%) reported other mental health diagnoses with most reporting no (33.8%) or minimal (50.7%) interference from those difficulties. One third (33.3%) had sought help for an eating disorder and around 20% received prolonged/intensive treatment during the follow-up period. Approximately 70% had sought treatment for other mental health difficulties (mostly anxiety or depression) and 35.4% had substantial treatment. At follow-up more than half (55.5%) reported doing generally well, and around two-thirds reported general satisfaction with their social well-being (65%). The majority (62.7%) had a good outcome on the Morgan Russell criteria, which was consistent with low self-reported ratings on EDE-Q, and low impact of eating disorder or mental health symptoms on work and social engagement. Most of the former patients who had day and/or inpatient treatment as a part of their comprehensive integrated care at MCCAED did well at follow-up.

**Conclusions:**

Young people seen in specialist eating disorder services do relatively well after discharge at longer-term follow-up especially regarding eating disorders but less favourably regarding other mental health difficulties. Few reported a diagnosable eating disorder, and the great majority went on to perform similarly to their peers in educational and vocational achievements.

**Supplementary Information:**

The online version contains supplementary material available at 10.1186/s40337-022-00535-8.

## Plain English summary

The aim of this study was to evaluate the long term outcome of children and adolescents who had received evidence-based treatments for an eating disorder in a specialist eating disorders service. The study was designed in collaboration with former service users, to ensure that measures of recovery were relevant to people with lived experience. One-hundred-and-forty-nine former patients consented to participate and completed online questionnaires. Very few (6.7%) reported having an eating disorder at follow-up, however, more than half (53.6%) reported other mental health difficulties during the follow up period. One third sought help for an eating disorder and around 20% had prolonged and intensive eating disorder treatment. Approximately 70% sought help for other mental health difficulties and nearly a third had substantial treatment for these during the follow-up period. Regarding education and work, most participants were functioning equivalently to their peers. More than half (55.5%) reported doing generally well with respect to overall well-being, and around two-thirds reported general satisfaction with their social well-being and quality of life. The follow-up study suggests that most young people treated in specialist eating disorder services do relatively well at long-term follow-up, but many continue to need help with other mental health problems.

**Supplementary Information:**

The online version contains supplementary material available at 10.1186/s40337-022-00535-8.

## Background

Eating disorders are often characterised as disabling, chronic or relapsing conditions with high mortality rates [1, 2]. It is known that the risk of relapse is high [3], particularly for those treated as inpatients [4]. Follow-up studies of adolescents treated for AN and related disorders with eating disorder focussed family therapy, the first line recommended treatment by the National Institute for Health and Care Excellence (NICE) [5] and other national guidelines [6] show that a significant proportion continue to experience eating disorder symptoms at 30-month [7], 4-year [8, 9] and 5-year [10] follow-up. Less is known about the outcomes following treatment for BN in adolescence, with one study reporting less than 50% abstinence rates from the core BN binge/purge symptoms at 12 months follow-up post treatment [11].

Long term longitudinal studies of eating disorders at a population level with people who were adolescents in the 1970s indicate that while eating disorder symptomatology improves over time, impairments in mental state, psychosexual wellbeing and socioeconomic wellbeing remain at up to 18-year follow-up [12]. Recent studies at a population level [13] of a 6-year follow-up of 11–17 year olds indicate stability of eating disorder symptoms and associations with poor mental health outcomes.

Risk of relapse or chronicity has been reported for adolescents who had longer inpatient admissions or psychiatric comorbidity [14] and in several studies examining outcomes over 5–8 years after early onset anorexia in childhood, about a quarter of children are reported to have a poor outcome according to Morgan Russell criteria [15] at follow-up [16]. In a study of inpatient family-based treatment with a mean follow-up of four and a half years, 35% remained below a healthy body weight and only 36% were classified as fully recovered [17]. The presence of eating disorder symptoms at follow-up has been predicted by raised levels of maternal criticism and hospital admission [10], end of treatment body weight [8] in AN, and by abstinence from purging and reduction in restraint measured by the Eating Disorder Examination [8] in BN. At 20–25-year follow-up in a mixed sample of adult and adolescent onset eating disorders, depression was associated with ongoing AN, and longer duration of BN symptoms at baseline was associated with ongoing BN [18].

Despite these indications of ongoing eating disorder symptoms, there is also recent evidence that few people treated in childhood or adolescence with an evidence-based treatment later seek help for eating disorder related difficulties, with 68.3% not seeking treatment post-18 years [19]. Of those who did seek treatment, 13% had a brief period of treatment in adult services and only 10% had a longer or more intense course of treatment. The receipt of later treatment was predicted by an older age at treatment in child services and more intense use of child services.

There is discrepancy between findings of low treatment seeking behaviours over the age of 18 and a strong body of evidence for ongoing symptoms. This discrepancy may reflect better long-term outcome following treatment for a more recent sample treated naturalistically in the community without the constraints of trial protocol lengths and with more modern treatment strategies than some of the longer follow-up papers. Alternatively, it may reflect an under-estimation of the extent to which people continue to experience difficulties without seeking treatment.

Most follow-up studies that have examined a treated sample have focused on core eating disorder symptoms via self-report or interview and/or biometrics. In contrast, community-based research has indicated that a broader view of the sequalae of eating disorders is warranted. There has been considerable variety and lack of agreement within the field around definitions of remission and recovery [20, 21]. However, recent explorations of the conceptualisation of recovery have highlighted the need to look beyond eating disorder symptoms and biometrics and to include psychological well-being, self-resilience and autonomy [22–24].

It has also been seen that eating disorders in adolescence are associated with other behavioural risk related difficulties in young adulthood [25]. Moreover, in community samples followed up 10 years after the onset of eating disorder difficulties, half of the young people did not have ongoing difficulties with eating disorder behaviours, but there was a higher incidence of affective and obsessive–compulsive disorders than in a control sample [26]. Similarly, low levels of eating disorder diagnoses alongside high levels of depression and anxiety have been reported at 6-year follow-up in those who had BN as adolescents [27].

This study is a follow-up of a cohort of 357 children and adolescents treated in a specialist eating disorder service (Maudsley Centre for Child and Adolescent Eating Disorders, MCCAED) between 2009 and 2014 [28]. It aimed to delineate the outcomes of people who received treatment for an eating disorder in childhood or adolescence to further inform our understanding of the maintenance of treatment effects across a wider range of areas of functioning and to identify possible factors predicting long term outcome to inform future treatment development.

## Method

### Sample

The sample comprised former patients with an ICD-10 diagnosis of AN/Atypical AN or BN/Atypical BN who attended treatment at MCCAED between 1st August 2009 and 31st January 2014. Treatment characteristics and outcomes at discharge from the service are reported elsewhere for this sample [28].

### Treatment setting

MCCAED is a community-based child and adolescent eating disorders service within the UK National Health Service (NHS) providing specialist treatments for children and adolescents with eating disorders for an area of South East London with a population of approximately 2.2 million people. MCCAED provides a comprehensive evidence-based treatment programme, central to which is eating disorder focussed family therapy but also a range of other evidence-based treatments. Most of the treatment is provided on an outpatient basis but for those who require more intensive care, the service offers an intensive day treatment programme and access to paediatric and psychiatric inpatient care. Further details of the service and the treatments patients received within MCCAED are reported in a separate (Part 1) paper which includes end of treatment outcomes and details of the MCCAED care pathways [28].

### Procedure

This study used a longitudinal follow-up design. Former patients were approached using the last known contacts for them available via their medical records. Where they were not contactable via these details, their parents were contacted using the last known contact and asked to facilitate contact between their son/daughter and the research team. A total of four attempts to make contact via phone, email or letter were permitted. Former patients who consented to participate were sent links to online questionnaires to complete via Qualtrics [29], an online data collection platform.

### Research ethics and registration

Permission for the study was provided by the UK NHS Research Ethics Service (ref: 18/LO/2001). Permission to use details held in medical records was granted by the NHS Health Research Authority, Confidentiality Advisory Group (ref: 19/CAG/0004). The research was registered with Clinical Trials (clinialtrials.gov) ID NCT03946540 on 10 April 2019. https://www.clinicaltrials.gov/ct2/show/record/NCT03946540

### Measures

#### Eating disorder examination questionnaire (EDE-Q)

The EDE-Q [30] is a 28-item self-report measure of eating disorder symptoms comprising four subscales and a total score. It has been seen to have good psychometric properties [31]. An EDE-Q global cut off score of 2.09 for AN/Atypical AN and 2.62 for BN/Atypical BN was used here to indicate if participants were likely to have eating disorder symptoms within a clinical range [32]. Internal consistency is reported to be high (α > .90) for the EDE-Q with children and adolescents [33]. In the current study the Cronbach alpha demonstrated good internal consistency (global score α = 0.97).

#### The work and social adjustment scale (WSAS)

The WSAS [34] is a self-report measure of the impact of illness symptoms on work and social functioning. It is a five-item questionnaire with a total score range of 0–40 where higher scores indicate greater impairment. The WSAS has been demonstrated to have good psychometric properties, to identify constructs related to functioning rather than general impairment [35], and has been used with adults with eating disorders [36]. In the current study internal consistency was high (α = 0.91).

#### Study specific questionnaire

A study specific questionnaire was administered to gather information on symptoms, treatment, social and economic functioning and well-being since discharge. This was developed in collaboration with former service users, who were not part of the study, to ensure that it included items assessing features of recovery relevant to people with lived experience. The former patients consulted suggested adding questions on general and social well-being and on close others’ opinions of former patients’ weight and eating behaviours. Body mass index (BMI) was calculated from self-reported weight and height, provided by participants.

Data on employment was classified following the UK Standard Industrial Classification [37]. Data on highest qualification attained was classified following the UK government classification [38]. Data is reported according to the proportion of the sample for whom it would be possible for that level of education to have been achieved given their age at the time of follow-up.

#### Morgan Russell global scores

Outcomes at follow-up were coded following the modified Morgan Russell Criteria [15, 39] and drawing on information provided via self-report using the following criteria:

- Good—BMI ≥ 18.5 kg/m^2^, menstruation and no bulimic symptoms
- Intermediate—BMI ≥ 18.5 kg/m^2^, no periods or BN symptoms < 1 per week over the last month
- Poor—BMI < 18.5 kg/m^2^ or BN symptoms ≥ 1 per week over the last month.

For those who did not report a specific weight and height at follow-up we used the response to the question “Is your current weight considered by others to be in the healthy range?”. We checked how closely the response to this question corresponded with reported BMIs when both information was provided. Of those who had a BMI below 18.5 kg/m^2^ (n = 17), 13 (76.5%) reported that others considered their weight as unhealthy. Three of the four with a BMI under 18.5 kg/m^2^ who said that others would consider their weight to be healthy were menstruating. Similarly, of the 52 with a reported BMI between 18.5–25 kg/m^2^ only 2 (3.8%) said that others considered their weight to be unhealthy (both had a BMI of 18.6 kg/m^2^, one was amenorrhoeic and both also expressed their own concern about being underweight). Five participants had a BMI > 25 kg/m^2^ at follow-up and three of those said that others considered their weight to be unhealthy.

Outcomes at MCCAED discharge were coded as above, but with data extracted from clinical notes and with 85%mBMI thresholds.

#### Received treatment enhancement during MCCAED treatment

Patients with AN/Atypical AN were classified according to whether they were treated solely in the outpatient clinic (OPT) or had outpatient treatment plus Additional Intervention and Management (AIM) through day- or in-patient treatment. Further details can be found in the Part 1 paper [28].

#### Self-report measures from MCCAED treatment

Questionnaires completed at assessment and discharge from MCCAED include the EDE-Q [30], Moods and Feelings Questionnaire (MFQ) [35–37, 40] and Screen for Child Anxiety Related Disorders (SCARED) [41]. Scores from self-reported versions of EDE-Q, SCARED and MFQ at assessment and discharge were used here to compare sample characteristics. All have demonstrated good psychometric properties. Further details have been reported in the Part 1 paper [28].

### Analysis plan

The primary analysis of the data is a descriptive analysis of key health and socio-economic outcomes at follow-up. Four sets of subsequent inferential analysis are reported. First, the relationship between ED symptoms and functioning is examined though correlational analysis of the EDEQ and WSAS. WSAS data were analysed via repeated measures ANOVA to identify particular areas of impairment. Second, chi squared analyses were conducted to analyse the maintenance of end of treatment outcome at follow-up according to adapted Morgan Russell outcome criteria at each time point. Third, Kruskal Wallis analyses were conducted on BMI, EDEQ and WSAS data to examine the extent to which these varied with outcome classified by adapted Morgan Russell criteria. Fourth, a series of ANCOVA and chi-squared tests were conducted to analyse the extent to which follow up outcomes varied with need for earlier treatment enhancement as described above.

A secondary analysis had been planned and registered to predict who continues to require mental health care following discharge from MCCAED. Power was calculated following advice for power calculation for logistic regression of 13 events (continuing mental health care needs) per variable [42]. Given the final sample size, the proportion of young people requiring mental health care and reporting on-going eating disorder symptoms did not allow for the planned analysis to be conducted.

### Impact of COVID-19 pandemic

During data collection, on the 23rd of March 2020, the UK entered national lockdown in response to the COVID-19 pandemic. In response to this, participants contacted during this period (n = 15) were asked to respond to the EDE-Q and WSAS contemporaneously and ‘as if’ they would have done in February 2020. A sensitivity analysis was conducted between these scores. There was no significant difference in the EDE-Q or WSAS score pre COVID-19 and contemporaneous scores. Data substitution into analyses of these scores did not alter the direction or significance of results reported below using contemporaneous scores. We therefore report only the latter scores.

## Results

### Sample

**Fig. 1:**
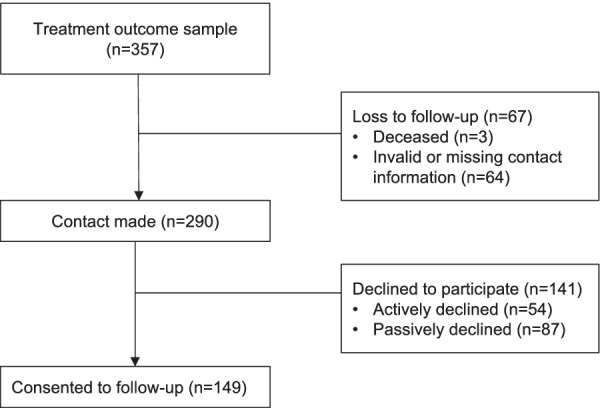
Flow chart of former patients’ follow-up recruitment

Of the 357 former patients who were eligible for this follow-up study, 64 had invalid or missing contact information and 3 were deceased. Contact was made with the remaining 290 (81.2%). Of these, 149 consented to participate. From the remaining 141 who did not take part in the study, 54 actively refused to participate. Initial contact was made with the remainder (n = 87); however, they were unreachable or did not respond to correspondence thereafter (maximum three follow-up attempts at contact via telephone or email). Retention rate to follow-up was 41.7% of the total eligible sample and 51.2% of the contactable sample (see Fig. 1).

ICD-10 was used to diagnose the patients in this sample. Young people who presented with a fairly typical clinical picture of anorexia nervosa but for whom one or more of the key features were absent were diagnosed as having atypical anorexia nervosa. The two most common reasons to receive the diagnosis of atypical anorexia nervosa were not meeting either (a) the low weight criteria or (b) amenorrhea criteria. Very occasionally someone would receive the diagnosis due to a lack of fear of weight gain. Young people with a fairly typical picture of bulimia nervosa but with one or more key features absent were diagnosed as atypical bulimia nervosa. For most of them the frequency/pattern of binge/purge behaviours was below the criteria for bulimia nervosa. Young people with clinical presentations characterised by other eating difficulties (e.g. selective eating) were not given the diagnosis of an atypical eating disorder. See Table 1 for descriptive data for the follow-up sample and duration of follow-up.

**Table 1:** Descriptive data of follow-up sample (N = 149)

		n (%)
Gender	Female	143 (96.0%)
	Male	6 (4.0%)
Ethnicity	White British	126 (84.6%)
	BAME	23 (15.4%)
ICD-10 Diagnosis at assessment	AN/Atypical AN	119 (79.9%)
	AN	68 (45.6%)
	Atypical AN	51 (34.2%)
	BN/Atypical BN	30 (20.1%)
	BN	19 (12.8%)
	Atypical BN	11 (7.4%)
Treatment enhanced	OPT	116 (77.9%)
	AN/Atypical AN	87 (58.4%)
	BN/Atypical BN	29 (19.5%)
	AIM	33 (22.1)
	AN/Atypical AN	32 (21.5%)
	BN/Atypical BN	1 (.7%)
		Mean (SD)
Age at follow-up		23.39 (2.22)
Duration of follow-up		6.91 (1.24)

AIM, Additional Intervention Management (outpatient + day or inpatient treatment); AN, anorexia nervosa; BAME, Black, Asian, or Minority Ethnic; BN, bulimia nervosa; OPT, Outpatient treatment only; SD, standard deviation

Analysis of difference in key variables between those who consented to follow-up and those who declined or were lost to follow-up revealed that people from a Black, Asian or Minority Ethnic (BAME) background were significantly less likely to consent to follow-up (χ^2^ (1, n = 357) = 7.15; *p* = .007). Those who consented to follow-up had higher self-reported MFQ (t (300) = 2.88; *p* = .04) and EDE-Q (t (303) = 2.08; *p* = .04) scores at the initial MCCAED assessment. There were no other significant differences between those who consented to follow-up and those who were not available to follow-up (See Additional file 1: Table S1).

### Follow-up diagnoses and symptoms of eating disorder

Participants reported few current diagnoses of eating disorders (n = 10, 6.7%) at follow-up. Of these, four reported diagnoses of AN, five of BN and one Atypical BN. Mean EDE-Q global score was 1.91 (SD = 1.61).

### Participants previously treated for AN/Atypical AN

BMI was calculated from available self-reported weight and height for 74 (62.2%) of the AN/Atypical AN sample. The mean BMI was 20.3 kg/m^2^ (*SD* = 2.9). Mean EDE-Q global score for the AN/Atypical AN was 1.74 (*SD* = 1.55). From the data available 64.8% (70/108) were below the clinical cut-off at the time of follow-up.

The great majority (n = 88/119, 73.9%) reported that others considered their weight to be within a healthy range, 20 (16.8%) said they were significantly underweight and 3 (2.5%) said they were overweight or obese. Thirteen (10.9%) continued to have irregular or absent menstruation, which they did not attribute to another gynaecological cause. Concerns expressed by others about rules for eating were reported by 55 (46.2%), with 7 (5.9%) reporting significant concern. The majority of these were described as being ongoing restrictive eating (n = 35, 29.4%). Other reported concerns related to binge and/or purge patterns, anxiety around mealtimes, over-exercise, irregular meal patterns and weight fluctuation.

### Participants previously treated for BN/Atypical BN

Compensatory behaviours including vomiting, laxative use and over exercise were reported by 43.3% (n = 13). These were less than weekly for 23.3% (n = 7), and more frequent for 16.7% (n = 5). Mean EDE-Q global score for the BN/Atypical BN was 2.62 (SD = 1.69). From the data available 50.0% (n = 13/26) were below the clinical cut-off at the time of follow-up. Concerns from others about rules for eating were reported by 43.3% (n = 13, of which 13% (n = 4) reported significant concerns) and restrictive eating by 23.3% (n = 7).

### Mental health difficulties

Diagnoses of mental health difficulties made since discharge from MCCAED or persisting since treatment in MCCAED were reported by 53.7% (n = 80) of participants at follow-up. The majority of these were either depression (28.8%), anxiety (17.5%) or both (32.5%). A diagnosis of borderline personality disorder was reported by 6.3%. Small numbers of participants (n = 1–4, 1.3–5%) reported other diagnoses which included Autism Spectrum Disorder, Body Dysmorphia Disorder, Obsessive Compulsive Disorder, Post Natal Depression, Substance Misuse, Bipolar Disorder, Attention Deficit Hyperactivity Disorder, Psychosis and Gender Dysphoria. Around half (n = 42) of participants reporting mental health difficulties reported two or more diagnoses.

Self-harm data are available for 139 (93.3%)  participants in the sample. Eighty (57.6%) reported engaging in self-harm at one point in their life. Of those reporting engaging in self-harm, 71.3% did this during their treatment with MCCAED, whilst the remaining 28.7% commenced self-harm after discharge. Of the participants who experienced self-harm whilst in treatment with MCCAED (n = 57), 31 continued to self-harm after discharge, whereas 26 did not. Eleven people (7.9%) reported engaging in self-harm recently (within 3 months of their follow-up). Of these, eight had self-harmed while in treatment with MCCAED, whereas 3 had not.

### Clinical service use since discharge from MCCAED

#### Eating disorder related help seeking

**Fig. 2:**
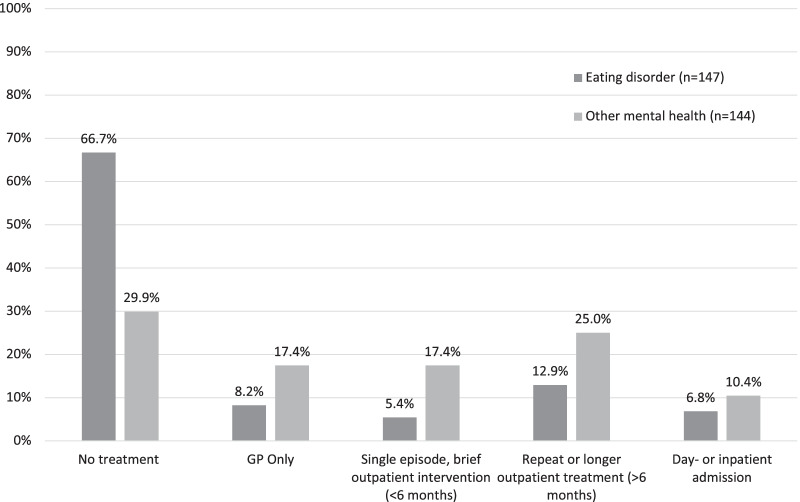
Treatments received during follow-up period

Engagement with any services for eating disorder treatment since discharge, was reported by 49 (33.3%) participants. Help was sought from a range of services including GPs, student services, charities, private therapy and specialist NHS services. Of the 98 (66.7%) who did not seek help, 28 (19.0%) reported not seeking help despite experiencing difficulties. The level of help received ranged from GP consultation or brief single engagement with outpatient services to more prolonged substantial treatment, defined here as 6 month or more of regular outpatient therapy or admission to day- or inpatient care. As can be seen in Fig. 2 which summarises the level of treatment received during the follow-up period, 29 (19.7%) participants received prolonged and more intensive treatments.

#### Mental health difficulties related help seeking

A somewhat different picture emerges when treatment for other mental health difficulties is considered with a total of 101 (70.1%) participants reporting receiving some level of help from services at some point during the follow-up period. Of the 43 (29.9%) who had no professional help, 20 (13.8%) had not sought help despite difficulties or were unable to find the right help. Again, the level of help received during the follow-up period varied and is summarized in Fig. 2. The need for substantial level of treatment for general mental health problems (most commonly anxiety or depression) was greater with 51 (35.4%) receiving such help.

#### Prolonged, repeated, and higher intensity treatments

During the follow-up period, 10.7% (n = 16) had substantial eating disorder treatment (> 6 months, or repeated, or day/inpatient admission(s)), 24.2% (n = 36) had substantial treatment for other mental health disorders (> 6 months, or repeated, or day/inpatient admission), and 10.1% (n = 15) had both substantial eating disorder and other mental health disorders treatments (> 6 months, or repeated, or day/inpatient admission). It should be noted that this includes those who continued treatment immediately following discharge from MCCAED, as 14.8% (n = 22) were discharged to adult eating disorder services for further treatment of eating disorders and 17.4% (n = 26) were discharged to community mental health services (CAMHS) for treatment of comorbidities.

### Well-being

**Fig. 3:**
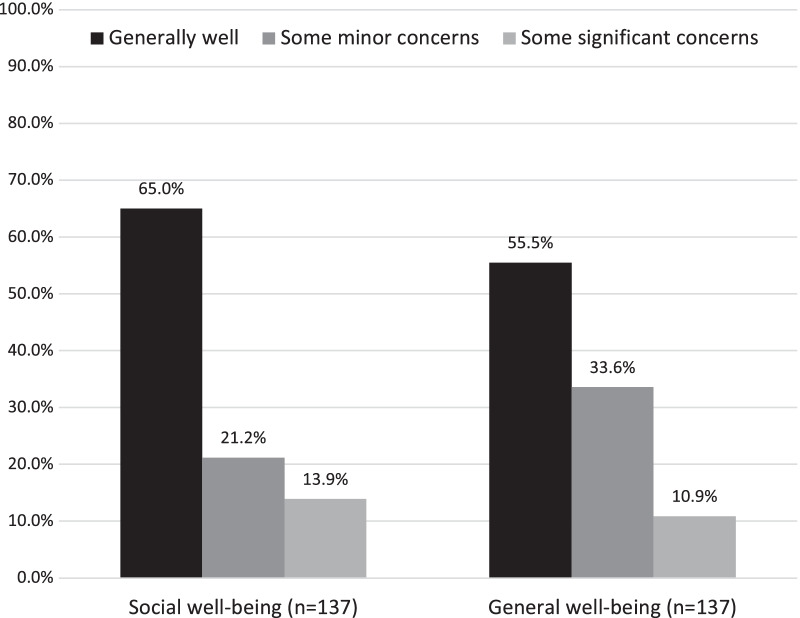
Self report of subjective social and general well-being at the time of follow-up

A broadly positive sense of well-being was reported by most of the participants with more than half reporting being generally well and nearly two thirds reporting general satisfaction with their social life (see Fig. 3). Seventy-six (69.1%) rated their quality of life as good or very good and only 9 (6.6%) rated it as bad.

### Functioning in education and work

Educational attainment was assessed according to the highest-level qualification that could be reached within each age group. Of the 18–20-year-olds (n = 20), who could have attained a level three qualification (equivalent to A Levels in the UK), 90% had done so. Of those who were older than 21 (n = 129), 47% had achieved a level 6 qualification (equivalent to an undergraduate university degree) or higher.

Thirteen participants (8.7%) reported that they were not in either education or employment. The remainder reported being in either education (n = 30, 20.1%), employment (n = 78, 52,3%) or both (n = 28, 18.8%). Fifty-four (36.2%) participants reported having had a break in their education or employment they attributed to difficulties with an eating disorder or other mental health difficulty.

### Living circumstances

Participants reported a range of living circumstances, with 42.3% living with parents, 13.4% owning their own homes, 2% in local authority housing and the remainder in either university accommodation or private rental.

### Impact of symptoms of eating disorders and other mental health difficulties

**Fig. 4:**
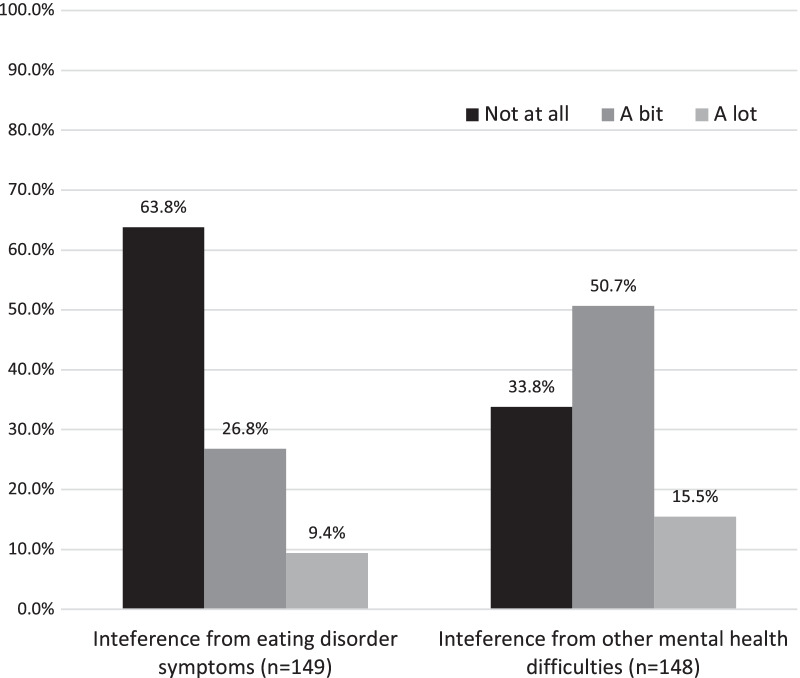
Do eating disorder or other mental health difficulties interfere with education or work

Figure 4 shows the self-reported impact of eating disorder and other mental health difficulties on work or education. The interference of ED symptoms varied and included management of symptoms, routines and treatment related commitments, as well as life decisions being influenced by proximity to support or treatment. Other mental health difficulties were reported to interfere through changes in mood, social engagement, lack of motivation and difficulties managing stress and/or workloads.

### The impact of eating disorder outcomes on work and social adjustment

Overall, the impact of eating disorder or mental health symptoms on work and social engagement (as measured by the WSAS) at the time of follow-up was low (WSAS mean = 8.62, *SD* = 8.98) but varied depending on the area of functioning (repeated-measures ANOVA—*F* (4, 528) = 7.39) *p* < .001). Planned deviation contrasts revealed significantly greater impairment on social leisure activities than other activities (mean leisure score = 2.14, *SD* = 2.30, *F* (1, 132) = 17.26, *p* < .001). WSAS scores significantly correlated with current EDE-Q global scores (*r* = .49, *p* < .001, n = 107), reflecting an association between higher levels of impairment on the WSAS and higher reports of current eating disorder symptomatology. This relationship remained significant when controlling for time since discharge.

Kruskal Wallis tests revealed significant differences in EDE-Q global score (*H*(2) = 26.35, *p* < .001) and WSAS total score (*H*(2) = 13.74, *p* < .001) between groups classified by Morgan Russell criteria. Post hoc pairwise comparisons of the groups revealed that both for the EDE-Q and WSAS the poor and intermediate groups did not differ from each other and that each was significantly different from the good outcome groups who reported fewer symptoms of eating disorders (Mdn = 0.71) and better work and social adjustment (Mdn = 9) (see Additional file 2: Table S2 for details).

### The maintenance of outcome between discharge from MCCAED and follow-up

Morgan Russell outcomes for the AN/Atypical AN sample at discharge from MCCAED and at follow-up are summarised in Table 2. For nine participants, data were missing not permitting us to rate Morgan Russell outcome at the follow-up. The majority (62.7% n = 69) of participants had a good outcome at follow-up. Sixty-eight percent (n = 41) of those with a good outcome at discharge from MCCAED maintained a good outcome. Of those discharged with an intermediate outcome at discharge from MCCAED, 60% (n = 14) had improved to good outcome and of those discharged from MCCAED with poor outcome, 51.8% (n = 14) had improved to a good outcome at the point of follow-up. Eighteen (16.4%) former patients had relapsed from a good or intermediate outcome to a poor outcome. This reduction in outcome was not typically due to weight loss, as the majority of this group (n = 16, 88.9%) had maintained a BMI above 18.5 kg/m^2^. Rather, the poorer outcome was due to bingeing (n = 9, 50.0%), vomiting (n = 4, 22.2%) or both (n = 3, 16.6%). In other words, while the majority with a worse outcome at follow-up would not merit a diagnosis of AN or Atypical AN, some would meet criteria for BN, Atypical BN or binge eating disorder (BED).

**Table 2:** Tabulation of change in Morgan Russell outcome assessment between discharge from MCCAED and follow-up for the AN/Atypical AN group

	Follow-up outcome			
	Good	Intermediate	Poor	
EOT outcome				
Good	41	7	12	60 (54.6%)
Intermediate	14	3	6	23 (20.9%)
Poor	14	3	10	27 (24.6%)
Total	69 (62.7%)	13 (11.8%)	28 (25.5%)	110

EOT, end of treatment in MCCAED

In the good outcome group on Morgan Russell criteria at discharge from MCCAED, 78.8% (n = 63) did not have any treatment for eating disorder beyond GP, and in the intermediate group 80.0% (n = 24) did not have any treatment beyond GP during the follow-up period. However, the rate was lower in the poor outcome group with only 62.1% (n = 18) not having any further treatment for their eating disorder.

Due to the relatively small sample size of the BN/Atypical BN group, a similar descriptive analysis of the BN/Atypical BN data are not reported.

### The need for additional intervention management during MCCAED treatment and outcome at follow-up

The AN/Atypical AN group who received a treatment enhancement with day program and/or inpatient admission during their treatment with MCCAED (AIM group) were more likely to have an episode of substantial treatment for eating disorder difficulties during the follow-up period (χ^2^ (1,119) = 3.99, *p* < .05) (see Table 3). However, there were no significant differences between the AIM and OPT group with regard to the likelihood of receiving treatment for mental health difficulties during the follow-up period, severity of eating disorder symptoms, general and social well-being at the follow-up, BMI or menstrual status.

**Table 3:** Enhancement of treatment during MCCAED treatment on follow-up measures

	OPT	AIM	
Follow up measure	Mean (*SD*)	Mean (*SD*)	Test statistic
*BMI*	20.68 (2.67)	19.18 (3.34)	*F*(1, 72) = 3.90, *p* = .052, *d* = .50
*EDE-Q global scores*	1.68 (1.53)	1.92 (1.63)	*F*(1, 106) = 0.50, *p* = .48, *d* = .16
*WSAS scores*	7.96 (9.20)	10.44 (9.42)	*F*(1, 104) = 1.00, *p* = .32, *d* = .27

	N (%)	N (%)	
*Menstrual Status*			
Irregular Periods/amenorrhea	8 (7.2%)	7 (25.0%)	χ^2^ (1, 104) = 3.47, *p* = .06
Regular periods/other	68 (92.8%)	21 (75.0%)	
*Further ED treatment*			
No or single brief intervention	74 (84.2%)	22 (68.8%)	χ^2^ (1, 119) = 3.99, *p* = .046
Repeat or more than 6-month intervention (incl. day or inpatient)^a^	13 (15.8%)	10 (31.3%)	
*MH difficulties*			
MH diagnoses	46 (53.5%)	17 (53.1%)	χ^2^ (1, 115) = 0.23, *p* = .63
No MH diagnoses	40 (46.5%)	15 (46.9%)	
*Further MH treatment*			
No or single brief intervention	57 (67.1%)	20 (64.5%)	χ^2^ (2, 116) = 3.27, *p* = .16
Repeat or more than 6-month intervention	21(24.7%)	5 (16.1%)	
Day or inpatient treatment	7 (8.2%)	6 (19.4%)	
*General well-being*			
Generally well	51 (61.4%)	13 (48.2%)	χ^2^ (2, 110) = 3.90, *p* = .14
Some minor concerns	25 (30.2%)	8 (29.6%)	
Some significant concerns	7 (8.4%)	6 (22.2%)	
*Social well-being*			
Generally well	58 (69.9%)	16 (59.3%)	χ^2^ (2, 110) = 1.15, *p* = .56
Some minor concerns	15 (18.1%)	6 (22.2%)	
Some significant concerns	10 (12.0%)	5 (18.5%)	

Enhancement of treatment: OPT/AIM analysis conducted on AN/Atypical AN sample only

^a^‘Repeat or more than 6-month intervention’ and ‘day or inpatient admission’ categories combined for analysis due to low frequencies

## Discussion

This 7-year follow-up study provides a description of the mental health, physical health and well-being outcomes of a sample of young people who received evidence-based treatments for their eating disorder in a specialist community-based child and adolescent eating disorder service (MCCAED).

The first aim of this study was to assess and describe persisting difficulties with eating disorder and comorbid symptoms, well-being and psycho-social functioning at longer term follow-up. Our findings emphasise the multifaceted nature of eating disorder recovery and highlight that the metric used to measure outcome very much influences how ‘well’ someone is considered to be functioning. On the one hand, there were very low levels (6.7%) of self-reported eating disorder diagnoses at the time of follow-up. The vast majority of participants reported satisfactory or greater quality of life (93.4%), low levels of significant interference of eating disorder difficulties on work and education (9.4%) and a low impact of eating disorder symptoms on work, social engagement and relationships at the time of follow-up. Overall young people’s work, education and housing were broadly in line with the UK national data [43–46]. In general, despite the presence of some eating disorder and comorbid difficulties for a third of the group, only 10% report significant interference in their day-to-day functioning. This suggests that a persistence of some symptoms does not necessarily impact on well-being. Arguably, this could make this group more vulnerable to relapse, however, more data is required to determine this.

On the other hand, using other metrics, the story could be interpreted somewhat differently, with approximately a third of participants struggling in various ways at follow-up. Self-reported eating disorder symptoms persisted for some, with approximately a third (35.2%) of those with AN/Atypical AN and half (50.0%) of those with BN/Atypical BN at discharge from MCCAED, reporting eating disorder symptoms above the clinical cut-off on the EDE-Q at follow-up. Likewise, concerns from others about eating behaviours and rule-following were reported by nearly half (43.3%), although only 5.9% of these were reported to be significant. This fits with general functioning data, which suggested that 55–66% reported being ‘generally well’ and were satisfied with their social well-being.

Somewhat unexpectedly, results of this follow-up demonstrated that more than half of participants reported another mental health diagnosis since discharge or persisting since receipt of treatment in MCCAED. Rates of self-harm were relatively high, with 57.6% of the sample engaging in self-harm at some point in their life, although only 16.5% commenced self-harm after discharge from MCCAED. Furthermore, two thirds reported interference from other mental health difficulties on their work and education, to some extent.

In comparison to previous reports [36], young people in this study reported higher levels of interference in daily life than previously reported healthy controls, but lower than those with active AN. Consistent with this, those with higher self-reported symptoms of eating disorders at follow-up also reported higher levels of interference from symptoms in work and social activities. However, mental health difficulties were thought to interfere more in work and education than eating disorder symptoms. For the majority of participants these were either depression or anxiety. This is higher than would be expected from national prevalence data. The Adult Psychiatric Morbidity Survey [46] indicates that symptoms of common mental health difficulties (depression and anxiety) are reported by 26% of 16–24-year-old women and 9.1% of men in the same age group. Again, this is consistent with previous reports [25, 26] of ongoing non-eating disorder difficulties at follow-up.

Findings from the current study are broadly consistent with previous longer-term follow-up data or better [47–49] although such comparisons are hampered by lack of consensus in the field on how to define recovery [21, 49]. In previous studies remission rates at follow-up range from approximately 30% to 85% depending on the treatment received, study setting and length of follow-up, and definitions of remission [48]. Similarly, other studies that have used the Morgan Russell criteria indicate 27–58% have a good outcome (62.7% in current study), 13–25% intermediate (11.8% in current study), 11–42% poor (25.5% in current study), and 1–11% (0.8% in current study) are deceased at 6–12 year follow-up [47].

The Morgan Russell criteria has its limitations and was used in this study primarily for the purpose of comparing outcomes with previous trials. Despite the limitations, the current study shows that those with a good outcome at follow-up report functioning significantly better on almost all measures of well-being. This suggests that a good outcome on the Morgan Russell criteria (i.e. weight above a minimum BMI threshold of 18.5 kg/m^2^ plus regular menstruation and an absence of binge/purge symptoms) may be indicative of well-being and improved functioning in a range of domains. However, the current data also suggest that those with an intermediate outcome on the Morgan Russell criteria appear to be functioning more closely to those with a poor outcome. In other words, long-term ongoing menstrual irregularities and/or occasional binge/purge behaviours, even when a minimal weight threshold has been reached, indicate that general well-being and psychosocial function are likely to be impaired.

A second aim of this study was to assess whether treatment outcomes were maintained at follow-up. The majority (68.3%) of young people who were discharged from MCCAED with a good outcome on the Morgan Russel continued to have a good outcome at follow-up. Additionally, around 60% of those classified as having an intermediate outcome, and more than half (51.8%) of young people who had a poor outcome at discharge from MCCAED had improved to a good outcome at follow-up. Still, 25.5% of young people were classified as having a poor outcome at follow-up. Of note, the majority of patients with AN/Atypical AN with a poor outcome at follow-up, would not merit a diagnosis of AN or Atypical AN, but a number would meet criteria for BN, Atypical BN or binge eating disorder (BED).

The level of treatment seeking in the current sample during the follow-up period is particularly striking. A third (33.3%) sought treatment for eating disorder difficulties and over two thirds (70.1%) for comorbid difficulties during the follow-up period. Furthermore, approximately a third of young people had substantial treatments (outpatient treatment > 6 months, repeated, or inpatient/day care) for the other mental health disorders. For many, this occurred more than four years after discharge from MCCAED, potentially suggesting an ongoing, persisting or reoccurring nature of mental health difficulties. Even those who do relatively well regarding eating disorder difficulties, may need additional ongoing support for other mental health difficulties for some time.

The final question explored in this study was whether those who received treatment enhancement (day and/or inpatient treatment) within the envelope of a comprehensive service model of integrated care with MCCAED, had different outcomes at follow-up compared with those treated purely as outpatients. Previous studies have shown that patients with AN treated in inpatient settings have worse outcomes to patients treated only in outpatient settings [48]. Direct comparisons between outpatient and inpatient/day treatment through randomised studies are fraught with difficulties [50, 51] and it is therefore difficult to know if the differences are due to patient/illness factors or treatment/service level factors. In the current study there were no significant differences at follow-up in rates of eating disorder symptoms and global and social functioning between patients who had additional day and/or inpatient treatment in MCCAED (AIM group) and patients who had only outpatient treatment. Patients in AIM group had a slightly lower BMI at follow-up though this difference was not statistically significant. The AIM group was, however, more likely to have a prolonged outpatient or day/inpatient treatment for ED during the follow-up period, which is consistent with reports from other studies of young people who have received inpatient treatment [17]. Together this suggests that those who were arguably more unwell during MCCAED treatment, received more intensive support, but ultimately, with the additional treatment, the majority did well.

### Limitations

There are several, important limitations to the current study. Firstly, only 42% of the original service evaluation sample, and 51% of the contactable sample, consented to participate in this study. This raises questions as to how representative the findings are of the whole sample, making interpretations tentative.

Despite the sample size, the sample itself was broadly clinically representative of the patients treated in MCCAED. The sample was slightly higher in self-reported mood symptoms and eating disorder symptoms at assessment in MCCAED, and while these differences were not seen at discharge from MCCAED, it is possible that these differences reflect a group of young people who may have been more vulnerable to the subsequent mood related difficulties and self-reported interference from eating disorder symptoms which they report at follow-up.

The sample is less representative in terms of ethnicity, with a lower proportion of people of Black and Asian ethnicity participating in the follow-up. This limits the conclusions that can be drawn regarding outcomes from eating disorders in adolescence in the general population.

The findings are also limited by the self-report nature of this research. While this maximised the number of young people that could be reached at follow-up, self-report questionnaire data may be less reliable than a standardised clinical interview.

Lastly, a secondary aim of this research was to examine potential predictors of outcome at follow-up. This was precluded by the fact that only 51.4% of former patients consented to participation in this study. This highlights the need for prospective follow-up of naturalistic samples which allows for early and ongoing engagement in the research process, and management of missing data through modelling across various time points. The low numbers in the current sample preclude data imputation, for example, which would have been possible had there been multiple sampling points.

## Conclusions

This research highlights that young people seen in community-based specialist eating disorder services do relatively well after discharge at longer-term follow-up. The majority report adequate or good quality of life, few report a diagnosable eating disorder, and many go on to perform similarly to their peers in educational and vocational achievements. This is irrespective of whether or not they received additional day and /or inpatient treatment whilst in MCCAED. Encouragingly, half of those (50%) who were discharged from treatment by MCCAED with a poor outcome, went on to have a good outcome, highlighting that even those who present with more severe or complex difficulties can do well in the longer-term. The current data also highlights that, while some eating disorder and comorbid symptoms may persist, only a small minority report these to interfere significantly in day-to-day life. Indeed, it is a strength of this paper that the research has examined functional outcomes beyond ED symptoms, an area that has been highlighted by service users as vital [23, 24]. Nevertheless, treatment seeking during the follow-up period was notable, with a third going on to engage in further treatment for eating disorder related difficulties and two-thirds for other mental health difficulties.

Together, these findings suggest that some eating concerns may persist beyond treatment, however, they are not at a level that significantly impacts functioning and well-being for the great majority. The current findings support the recent moves in the field for treatments (and their research evaluation) to incorporate a broader focus on emotional well-being and functioning, not just eating disorder difficulties. However, the relatively high rates of treatment seeking raises questions as to whether comorbid difficulties need to be addressed more actively in current eating disorder treatments, which has implications for future treatment and service development.

## Supplementary Information

**Additional file 1**. Comparison of those who consented to follow-up and those who were not available to follow-up.

**Additional file 2**. Breakdown of total EDE-Q and WSAS scores at follow-up by Morgan Russell outcome categorisation.

## Data Availability

Data are available from the corresponding author on reasonable request.

## Abbreviations

AN: Anorexia Nervosa
%mBMI: Percentage median body mass index
AIM: Additional intervention and management group
ANOVA: Analysis of variance
ANCOVA: ANALYSIS of covariance
BAME: Black, Asian or Minority Ethnic
BED: Binge Eating Disorder
BMI: Body mass index
BN: Bulimia nervosa
CAMHS: Child and adolescent mental health service
ChOCI: Children’s Obsessional Compulsive Inventory
COVID-19: Coronavirus disease
ED: Eating disorder
EDE-Q: Eating Disorder Examination Questionnaire
GP: General practitioner
MCCAED: Maudsley Centre for Child and Adolescent Eating Disorders
Mdn.: Median
MFQ: Mood and Feelings Questionnaire
MH: Mental health
NEET: Not in either education or employment
NHS: National Health Service
OPT: Outpatient treatment group
SD: Standard deviation
SPSS: Statistical Package for Social Sciences
UK: United Kingdom
WSAS: Work and Social Adjustment Scale
