# Supplementary material for: From efficacy to effectiveness: child and adolescent eating disorder treatments in the real world (Part 2): 7-year follow-up
Source: J Eat Disord. 2022 Feb 5;10:14. doi: 10.1186/s40337-022-00535-8 (PMC8817149; doi:10.1186/s40337-022-00535-8)
Supplement: Supplementary file 1 — Additional file 1. Comparison of those who consented to follow-up and those who were not available to follow-up. [file 40337_2022_535_MOESM1_ESM.pdf]

		Follow up sample n (%)	*Uncontactable or declined n (%)*	*Test Statistic*
Number in sample		149	208	
Gender	Female	143 (96.0%)	189 (90.8%)	1, 357) = 3.78,
	Male	6 (4.0%)	19 (9.1%)	p = .06
Ethnicity	White British	126 (84.6%)	151 (72.6%)	**χ^2 (1, 357) = 7.15,**
	BAME	23(15.4%)	57 (27.4%)	***p* = .007**
Diagnosis	AN	68 (45.6%)	77 (37.0%)	χ^2 (1, 357) = .313,
	Atypical AN	51 (34.2%)	94 (45.2%)	*p* = .57[b]
	BN	19 (12.8%)	23 (11.1%)	
	Atypical BN	11 (7.4%)	14 (6.7%)	
Treatment enhanced[a]	OUT	87 (73.1%)	126 (73.7%)	χ^2 (1, 290) = 0.01,
	AIM	32 (26.9%)	45 (26.3%)	*p* = .91
	Psychiatric admission	5 (3.4%)	7 (3.4%)	
	ITP	19 (12.8%)	24 (11.2%)	
	Admission and ITP	9 (6%)	17 (8.25)	

		n	*Mean (SD)*	*95% C.I.*	*n*	*Mean (SD)*	*95% C.I.*	
Age	At discharge	149	16 yrs 5.3 mths (1 yr, 7.3 mths)	16yrs 1.9 mths - 16yrs 8.3 mths	208	16yrs 1.5mths (1yr, 10.8 mths)	15yrs 10.4mths - 16yrs 4.5mths	*F* (1, 355) = 2.64, *p* = .10 *d = 0.18*
	At follow up	149	23 yrs 3.1 mths (2 yrs 2.9 mths)	22yrs 10.8 mths - 23yrs 7.5mths	201	22yrs 11.8 mths (2yrs 1.8 mths)	22 yrs 8.2 mths – 23yrs 3.2mths	*F* (1, 348) = 1.36, *p* = .24, *d =-0.13*
MCCAED measures	EDE-Q (global) assessment	132	3.54 (1.64)	3.25 – 3.82	173	3.13 (1.73)	2.87 – 3.39	***F* (1, 303) = 4.34, *p* = .04, *d* = -0.24**
	EDE-Q (global) discharge	59	1.81 (1.61)	1.39 – 2.23	84	1.49 (1.57)	1.15 – 1.83	*F* (1, 141) = 1.39, *p* = .24, *d = -0.20*
	MFQ assessment	129	33.75 (16.47)	30.88 – 36.61	173	28.37 (15.73)	26.02 – 30.74	***F* (1, 300) = 8.27, *p* = .004 *d* = -0.34**
	MFQ discharge	57	16.61 (16.03)	12.36 – 20.87	84	18.59 (16.11)	15.10 – 22.09	*F* (1, 139) =0.51, *p* = .47, *d = 0.12*

	SCARED assessment	172	28.77 (19.68)	25.38 – 32.16	132	28.29 (17.11)	25.72 – 30.87	F, (1, 302) = 0.05, *p* = .82, *d* = *-0.03*
	SCARED discharge	59	22.20 (17.39)	17.67 – 26.73	82	21.09 (15.71)	17.63 – 24.54	F, (1, 139) = 0.16, *p* = .69, *d* =*-0.07*
MCCAED biometrics	%mBMI assessment	147	85.90 (12.25)	83.90 – 87.89	205	86.72 (15.05)	84.65 – 88.79	F, (1, 350) = 0.30, *p* = .59, *d* =*0.06*
	%mBMI 3 months	130	88.85 (10.48)	87.04 – 90.67	176	89.35 (11.48)	87.64 – 91.05	F, (1, 304) = 0.15, *p* = .70, *d* = *0.05*
	%mBMI discharge	143	93.86 (11.22)	92.00 – 95.71	201	94.63 (13.84)	92.70 – 96.55	F,(1, 342) = 0.30, *p* = 0.59, *d* = *0.06*

Supplementary Table S1 | Comparison of those who consented to follow-up and those who were not available to follow-up

[a]AN/Atypical AN cases only
[b] Comparison between AN/Atypical AN and BN/Atypical BN groups

Abbreviations: %mBMI, percentage of median body mass index AIM, Additional Intensive Management (day treatment or inpatient treatment; AN, anorexia nervosa; BAME, Black, Asian or Minority Ethnic: BN, bulimia nervosa; EDE-Q, Eating Disorder Examination Questionnaire global score; MFQ, Mood and Feelings Questionnaire; OPT, Outpatient treatment only; SCARED, Screen for Child Anxiety Related Disorder; SD, standard deviation
