# Supplementary material for: From efficacy to effectiveness: child and adolescent eating disorder treatments in the real world (Part 2): 7-year follow-up
Source: J Eat Disord. 2022 Feb 5;10:14. doi: 10.1186/s40337-022-00535-8 (PMC8817149; doi:10.1186/s40337-022-00535-8)
Supplement: Supplementary file 2 — Additional file 2. Breakdown of total EDE-Q and WSAS scores at follow-up by Morgan Russell outcome categorisation. [file 40337_2022_535_MOESM2_ESM.pdf]

	Morgan Russell Outcome Score	n	Mean (SD)	Median (IQR)	Kruskal Wallis test		Pairwise comparisons		
					H	*p*	good - intermediate	good - poor	intermediate - poor
EDE-Q	good	67	1.12 (1.01)	0.71 (1.56)	26.35	$p < .001$	$p < .001$	$p < .001$	$p = .641$
	intermediate	13	2.81 (1.51)	2.76 (2.98)					
	poor	28	2.73 (1.87)	2.22 (3.36)					
WSAS	good	66	6.32 (8.60)	3 (9)	13.74	$p < .001$	$p = .028$	$p < .001$	$p = .765$
	intermediate	13	11.46 (8.77)	9 (13)					
	poor	28	12.61 (9.59)	12 (11.25)					

Supplementary Table S2 | Breakdown of total EDE-Q and WSAS scores at follow-up by Morgan Russell outcome categorisation

Abbreviations: EDE-Q, Eating Disorder Examination-Questionnaire global score; IQR, interquartile range; WSAS, Work and Social Adjustment Scale
